# Mediterranean Jellyfish Venoms: A Review on Scyphomedusae

**DOI:** 10.3390/md8041122

**Published:** 2010-04-04

**Authors:** Gian Luigi Mariottini, Luigi Pane

**Affiliations:** Dipartimento di Biologia, Università di Genova, Viale Benedetto XV 5, I-16132, Genova, Italy; E-Mail: pane@unige.it

**Keywords:** jellyfish, venom, Mediterranean Sea, Scyphomedusae

## Abstract

The production of natural toxins is an interesting aspect, which characterizes the physiology and the ecology of a number of marine species that use them for defence/offence purposes. Cnidarians are of particular concern from this point of view; their venoms are contained in specialized structures–the nematocysts–which, after mechanical or chemical stimulation, inject the venom in the prey or in the attacker. Cnidarian stinging is a serious health problem for humans in the zones where extremely venomous jellyfish or anemones are common, such as in temperate and tropical oceanic waters and particularly along several Pacific coasts, and severe cases of envenomation, including also lethal cases mainly induced by cubomedusae, were reported. On the contrary, in the Mediterranean region the problem of jellyfish stings is quite modest, even though they can have anyhow an impact on public health and be of importance from the ecological and economic point of view owing to the implications on ecosystems and on some human activities such as tourism, bathing and fishing. This paper reviews the knowledge about the various aspects related to the occurrence and the stinging of the Mediterranean scyphozoan jellyfish as well as the activity of their venoms.

## 1. Introduction

Several marine species produce harmful substances, which are used for defence/offence purposes and characterize the physiology and ecology of a number of organisms, among which cnidarians are particularly important. In general, a toxin is defined as “a substance (molecule) elaborated by a living organism that has an adverse effect on some living process” and venoms are “complex secretions that are composed of many active constituents, usually including a variety of toxins and accessory substances which facilitate the envenomation process” [1].

Cnidarian venoms are contained in specialized structures, called nematocysts, consisting of a capsule of proteinaceous nature containing a tightly wrapped and spiralized thread, which after mechanical or chemical stimulation is quickly extruded injecting the venom in the prey or in the attacker. The nematocysts are one of the three categories of cnidae, also called cnidocysts; a cnida discharges by eversion of its tubule [2].

In the Mediterranean region the studies concerning the biology, the ecology and the toxicology of jellyfish noticeably developed only during the last decades, mainly due to the proliferation phenomena occurring in several coastal zones. In particular, up until the 1970s the studies on the toxicity of Mediterranean jellyfish were scarcely of interest for human pathology owing to their weak toxic properties. Ever after, research was mainly aimed on the Scyphomedusae (Cnidaria: Scyphozoa) and particularly on Rhizostomoidea and Semaeostomeae (according to Avian [3]) that greatly sustained the bloom.

The Scyphozoan life cycle shows generally small polyps and sexual phases represented by quite large jellyfish provided with lobes on the marginal umbrella. According to Riedl [4], over 250 species of marine Scyphozoa are known and, among them, nearly 20 are known to live in the Mediterranean Sea and only 13 are common; Tregouboff and Rose [5] reported the occurrence of approximately 12 species of scyphozoan jellyfish in the Mediterranean Sea [6]. A recent classification [3] indicated that the large jellyfish of the class Scyphozoa belong to the subclass Scyphomedusae, which comprises three orders: (I) Stauromedusae, benthic and sessile, which resemble polyps and are uncommon in the Mediterranean Sea, (II) Coronatae, characterized by small polyps, often devoid of the medusoid phase, (III) Semaeostomeae and one superorder (Rhizostomoidea) subdivided into two orders (Cepheida and Rhizostomida), the large pelagic medusae, whose life cycle includes a small polypoid phase, difficult to find and to classify; in some species, such as *Pelagia noctiluca*, a polypoid phase is not known. Other indications about the taxonomy of scyphozoan jellyfish can be gathered from Franc [7] and from the European Register of Marine Species (http://www.marbef.org/data/erms.php). These jellyfish have gelatinous consistency; some of them can reach a diameter of 40 cm and over, are often characterized by intense colouring or can be transparent, are generally common in the open-sea and also near the coasts, where they can exert their irritant activity on bathers and sea-workers.

As the toxicity of these organisms is a well known subject [8–10], the aim of this paper is to review the knowledge about the venoms of the Mediterranean species and the impact they exert on humans and their activities. Even though the term “jellyfish” could have a wider meaning, including also some organisms other than scyphozoan medusae, such as hydromedusae, siphonophores and ctenophores [11], for the purposes of this article it will be utilized to indicate only the large pelagic scyphozoans living in the Mediterranean Sea that are pointed out as quite common [4,12]. These are the Semaeostomeae *Aurelia aurita*, *Chrysaora hysoscella* and *Pelagia noctiluca* and the Rhizostomoidea *Cotylorhiza tuberculata* and *Rhizostoma pulmo*, as well as the lessepsian species *Rhopilema nomadica*. Some news concerning the Coronata *Nausithoe punctata* and some other occasionally recorded jellyfish are also reported.

## 2. Cnidarian Jellyfish Venoms

Natural compounds produced by plants and animals have long been a source of substances with medicinal and therapeutic activity useful to treat different diseases [13]. The biotoxins, being substances which at sublethal doses show different pharmacological properties, can be included among these compounds.

The marine organisms, in spite of their abundance and variety, had a limited employment as a source of substances of pharmacological interest [14,15] until several marine compounds were recognized to exert an activity against human pathologies affecting the cardiovascular, endocrine, immune and nervous systems, to have an influence on infectious diseases or to be antiinflammatory, antiplatelet, antitumoral or cytotoxic [16–19]. Some of these compounds are currently in preclinical phase trials and/or in phase I and II clinical studies [20–22]. A list of the marine-based natural products of biomedical significance can be found at http://www.marinebiotech.org/dfsindex_alpha.html.

Some bioactive substances were discovered in cnidarians, such as prostaglandins (15R)-PGA2 in the gorgonian *Plaxaura homomalla* [23], the Palytoxin local anaesthetic and vasoconstrictive agent discovered in the zoanthid *Palythoa toxica* [24], Pseudopterosin [25], Sarcodictyns and Eleutherobin. Cytolytic and antitumoral substances have Also been found: for example, prostanoid compounds from the Anthozoan *Clavularia viridis* were shown to inhibit the growth of HL-60 leukemic cells [26], the incidence and growth of SNC tumors induced by *N*-Ethyl-*N*-Nitrosourea were shown to be affected by the crude venom of the scyphozoan *Cassiopea xamachana* [27], the growth of Ehrlich ascites tumors grafted in mice was inhibited by crude extracts of tissues from jellyfish and soft corals [28], Equinatoxin extracted from *Actinia equina* showed antitumoral activity on cultured cells [29], the Palytoxin was shown to induce ion currents (channels permeable to Na^+^ and K^+^ and slightly permeable to Ca2+, choline and tetramethylammonium) in mouse neuroblastoma cells [30].

Notwithstanding the discovery of bioactive compounds in Cnidaria, and the fact that they were indicated as early as the 1970s as a potential source of natural bioactive compounds of pharmacological concern useful to develop new drugs or biomedical materials [8], it should be emphasized that the hazard that cnidarians represent for humans is their most evident character.

Cnidarian venoms are contained in microscopic double-walled thick capsules, the nematocysts, produced by all–and only–cnidarians and secreted by the Golgi apparatus of nematoblasts, the cells specialized for this function [31,32]. The nematocysts contain a tightly spiralized and differently shaped thread, which according to the species, is provided with spines and with a basal enlarged portion known as the ‘shaft’ [33]; after mechanical or chemical stimulation, the thread is everted, injecting the venom contained into the capsule [34–36]. Several morphological types of nematocysts have been identified, also in a single species; thus the morphology of the capsule, thread and shaft has also a taxonomical concern [8,33,37].

Nematocyst discharge can be caused by several mechanical and chemical stimuli [36,38]. Recently, the stimuli which give rise to the discharge and the cell structure of cnidarian capsules have been reviewed, showing that cnidae can trigger either independently or under the influence of adjacent cells. The discharge could be caused by mechanical stimuli, with and without chemosensitization, and by vibrational frequencies; some surface compounds (mucins, N-acetylated sugars, proteins) were shown to sensitize the cnidocytes to mechanical stimuli and the mechanical stimulus from prey itself was seen to trigger the sensitized cnidocyte and cnidae discharge [39]. Each nematocyst can be used only once, because after discharge it is destroyed by adjacent cells [8].

The nematocysts essentially paralyse and kill the prey and therefore their function is linked to the carnivore predatory characteristics of Cnidarians, which feed mainly on zooplankton and small fish [2,32,36,40–43]. Furthermore, several marine organisms, such as nudibranch [44–47] and cephalopod [48] molluscs can prey Cnidaria and utilize their nematocysts to also cause damage to humans [8]; some turbellarians after ingestion can acquire nematocysts [47], which remain undigested and pass from the gut to the external body where they are used as a defence tool [49].

It is well known that cnidarian stinging is a serious health problem along some Asian and Australian coasts and in Pacific archipelagos [50–52], where extremely venomous jellyfish or anemones are common; these organisms can cause severe envenomations with extensive dermonecrosis, oedemas, diffused neurotoxicity, motorial and respiratory problems, cardiovascular symptoms, hypotension and also lethal cases in humans [53–56]. In particular, the cubomedusan *Chironex fleckeri*, the sea-wasp, can cause serious cardiotoxicity within few minutes and several deaths have been reported along the Australian coastline during the last century [57,58].

The effect of the contact with nematocysts is similar to a prick and is caused both by the mechanical penetration of the thread into the tissues and by the activity of mast cells; the injected substances irritate the nerve endings and cause the characteristic stiffness, swelling, itchiness and pain [59].

The poison of cnidarians depends on the stinging species, but in general the venoms are mainly composed of proteins, peptides and other substances of pharmacological concern [60]; for this reason, they can act on humans as antigens, therefore evoking a defence response by the immune system with consequent production of specific antibodies and activation of the “memory” phenomenon, as demonstrated by the response of laboratory animals to the venoms of *Chironex fleckeri*, *Chiropsalmus quadrigatus* and *Chrysaora quinquecirrha* [8]. In any case, peptidic sodium channel neurotoxins, cytolytic proteins and non-peptidic toxins have been discovered in Cnidaria [61].

The nematocysts of Mediterranean Cnidaria cause generally modest damage to humans, except in cases of hypersensitivity; for this reason, research about the venoms of these organisms has not developed since the 1970s and it only noticeably increased after the recent jellyfish blooms [9,10]. Table 1 summarizes the occurrence, the cases of envenomation and the poisonousness of the main Mediterranean scyphozoan jellyfish; the known nematocyst types for each species are also reported.

### 2.1. *Aurelia aurita*(Linnaeus, 1758)

The “moon jellyfish” *Aurelia aurita* is a whitish medusa with flattened bell, violet or pink margins provided with several tentacles and four oral arms with fringings in the lower portion; four half-moon-shaped gonads can be easily seen.

*Aurelia aurita* is a cosmopolitan species living in temperate and temperate-cold waters with temperatures varying from −0.5 °C to 30 °C, and carries out vertical migrations under the influence of light [65]. This jellyfish can be usually found along all coasts of the Mediterranean Sea and of the Black Sea [65,66]. During the jellyfish bloom of the 1970s–80s, swarms of *Aurelia aurita* were observed in Egypt waters, particularly in January, even though irregular outbreaks were recorded during summer along Alessandria beaches, and their abundance was correlated to wind action [98]; abundant swarmings were reported also in the years before the bloom [77]. This jellyfish was indicated to be one of the main occurring medusa in Southern Adriatic Sea and in Northern Ionian Sea [69]; in the Ligurian Sea, *Aurelia aurita* was the second most-sighted jellyfish after *Pelagia noctiluca* [73]. Even though *Aurelia aurita* was not the main species responsible for the Mediterranean bloom, its biomass was reported to increase more than 30-times during the 1980s [78]. *Aurelia aurita* occurs constantly in Tunisian waters where its presence seems to be linked mainly to eutrophic and low saline waters [87]; particular adaptations allow *Aurelia aurita* to survive and to increase in biomass under eutrophic conditions[99].

On the whole, *Aurelia aurita* is a strong predator of copepods and fish larvae [42,43,100,101] and reproduces rapidly, achieving high abundance and biomass [78]; *Aurelia* probably do not form scyphistomae where high sedimentation rates and appearance of H_2_S occurs [102].

Several studies aimed to increase the knowledge about the biochemical characteristics of *Aurelia aurita,* and its implication in the marine food web indicate that *Aurelia aurita* has a low content of organic matter [103]. Total lipids were reported to amount to 0.02% of the fresh tissue; this low total lipid content of *Aurelia aurita,* compared to that of the stinging species *Pelagia noctiluca*, induced the Authors [104] to correlate the lipid armature of tentacles to the intensity of the bioactions exerted by the jellyfish and, on this basis, to define *Aurelia aurita* a “non-stinging scyphozoan species”. Subsequent studies identified several sphingophosphonolipids in *Aurelia aurita* and indicated that total lipids range between 0.031 and 0.036% of fresh tissue [105].

Heterotrichous microbasic eurytele nematocysts and two atrichous isorhizas, “a”-atrichs and oviform polyspiras, common and similar in shape to those of the highly stinging medusae *Chrysaora quinquecirrha* and *Cyanea capillata*, have been observed in scyphistomae polyps of *Aurelia* [67,68]. Subsequent electron microscopy studies [106] showed that the nematocysts of *Aurelia aurita* are arranged in trasverse bands across tentacles, the cnidocytes are short and surrounded by stereocilia. In scyphopolyps of *Aurelia aurita*, a new nematocyst type (heterotrichous microbasic rhopaloid nematocysts), earlier included within the euryteles, but differentiated from these having two swellings on the discharged shaft, was identified [45]; heterotrichous microbasic rhopaloid heteroneme nematocysts and two types of homotrichous isorhiza haploneme nematocysts in the scyphopolyps and in the planulae as well as heterotrichous microbasic euryteles and isorhizas with subspherical capsules in adult medusae were also described [45]. Small a-isorhizas followed by rhopaloids dominated in planulae; the latter were 1–2 μm smaller than that of the medusae. In *Aurelia* polyps, the nematocysts, mainly a-isorhizas, were particularly abundant on the tentacle tips [45].

*Aurelia aurita* is commonly considered innocuous for man and it has been defined as “harmless jellyfish” [105], but it can be anyhow a trouble for bathers when it occurs in great numbers [107]. It was reported that the nematocysts of the moon jellyfish give weak and not irritable stinging, but could anyhow irritate thin or sensitive skin, eyes and lips [108] and produce a modest itchiness [109]; therefore, *Aurelia* cannot be considered harmless [108]. In addition, although tentacles of *Aurelia aurita* are short, the marginal bell is provided with batteries of ring-arranged nematocytes and also the oral zone is rich of nematocysts [65].

Extracts obtained from *Aurelia aurita* were shown to contain proteolytic enzymes and a resulted irritant for skin and eye of rabbit [110]. The venom from *Aurelia* was shown to effect isolated frog muscle, as a complete and irreversible block of indirectly and directly elicited muscle twitches and an irreversible depolarization of the muscle membrane, probably caused by an increase in membrane permeability to sodium ions [111].

In addition, tentacle extracts from *Aurelia* showed phospholipase A2 (PLA2) activity [62], which is known to be a major contribution to animal venoms and to cause several pharmacological and toxicological effects such as neurotoxicity, myotoxicity and haemolysis; PLA2 enzymes are present in several Cnidaria [112], both in nematocytes and in tissues [113], may be involved in physiological cell membrane lipid metabolism, in the irritation at the stinging site and in the enhancement of the haemolytic activity and can act alone or together with other proteins [114]. PLA2 can play a role both in the offense (for the capture and digestion of the prey) and in the defense activity of Cnidaria, and also in the local and systemic inflammation on humans [112].

The venom of *Aurelia aurita* contains potent lethal, demonecrotic, vasopermeability and hemolytic factors [62], but a dramatic difference has been reported concerning the dangerousness of *Aurelia* from different zones of the world: the Old World *Aurelia* (from the Red Sea) were stated to be more dangerous than New World ones (*Aurelia aurita* from Chesapeake Bay) [62], but it seems that *Aurelia aurita* is variable in its stinging potency: strong envenomations were reported in the Gulf of Mexico [63], in Australia, along the southeast coast of Florida [62] and in Israel [64].

A case of significant envenomation with local cutaneous lesions and immunospecific serum antibodies development was reported in a patient stung by *Aurelia aurita*; in particular, the stinging immediately caused local pain and piloerection, after few minutes urticaria and ulceration, 3–9 days later encrusted lesions and two weeks after hyperpigmentation; serological tests permitted to reveal a cross-reactivity, with development of antibodies, between the venom of *Aurelia aurita* and those of *Chrysaora quinquecirrha*, *Cyanea*, *Chironex* and *Physalia* [63].

*Aurelia aurita* from Mexican Caribbean caused dermotoxicity on human skin of volunteers who immediately after contact developed sharp local pain lasting 30 minutes, and three minutes after the contact suffered intense itching and vesiculopapular and erythematous eruptions lasting 10 days. Furthermore, extracts from tentacular margins of *A. aurita* killed II and III stage nauplii of *Artemia salina* after five-hour exposure and caused hemolysis of human, sheep and bovine erythrocytes; a protein able to cause tetanic reactions, total paralysis and death to crabs within three minutes was obtained through Sephadex G-200 chromatography [115]. Recently, *Aurelia aurita* caused organization problems in Spanish waters because the Authorities were alarmed for the potential decrease of tourism caused by the conspicuous occurrence of jellyfishes [116].

The enzymes contained into extracts from *Aurelia aurita* were seen to highly affect cancer cells and the different phases of the mitotic cell cycle, mainly the M an G0 phases. On the whole, cell inhibition was shown to increase at the transition from interphase to the mitotic phase and to reach the maximum in the resting phase, with inhibition of growth or induction of apoptosis 20–87% in liver carcinoma cells, 21–78% in colon carcinoma cells, 30–39% in myosarcoma cells, 38–94% in adenohypophyse carcinoma cells, 40–89% in skin carcinoma cells and 41–91% in kidney carcinoma cells, with an high statistical significance for colon carcinoma and mainly for myosarcoma cells; it was suggested that the treatment with jellyfish extract could induce breaking of ester bonds of DNA of the cancer cells [110] and the cytotoxic activity could be due to various enzymes able to cause apoptosis of treated cells [117].

Medusa extracts also highly inhibit breast adenocarcinoma, lung carcinoma and leukemia cells; breast adenocarcinoma cells showed growth inhibition varying from 23% (G1 phase) to 63% (G0 phase) with significant results for G2, M and G0 phases; *Aurelia aurita* extracts were cytotoxic for lung carcinoma cells and caused inhibition ranging from 26% (G1 phase) to 78% (G0 phase) with significant values for all cell cycle phases excepting G1; the inhibition of leukemia cells ranged from 28% (G1 phase) to 86% (G0 phase) and was significant for all cell cycle phases excepting G1 [118].

Recently, an antimicrobial peptide, which was named “aurelin”, was purified from the mesoglea of *Aurelia aurita*. The peptide, which shows partial similarity with defensins and with K^+^ channel-blocking toxins of sea anemones, exhibited antimicrobial activity against Gram-positive (*Listeria monocytogenes*, strain EGD) and Gram-negative (*Escherichia coli*, strain ML-35p) bacteria [119].

At last, it was reported that *Aurelia aurita* caused remarkable damage to fishing activities, particularly to set, trawl and gill nets, to aquaculture and to power plants [88].

### 2.2. *Chrysaora hysoscella* (Linnaeus, 1766)

*Chrysaora hysoscella* (“compass jellyfish”) is a yellowish medusa with brown-violet lines on bell surface. The bell is hemispherical and flattened; its margins carry 32 lappets and 24 tentacles. The oral arms are four, very long, with several fringings in the upper part and are scallop-edged in the lower part. Unlike other scyphozoan jellyfish, it is a protandric hermaphrodite species [65].

This jellyfish occurs in cold and temperate sea waters–in fact it can live at temperatures from 4 °C to 28 °C [65]–as well as in the Mediterranean Sea [75], and in upwelling areas such as in the Benguela ecosystem extending from northern Namibia to south of Cape Point in South Africa [120]. *Chrysaora hysoscella* is frequent in the Mediterranean Sea and occurs preferably in swarms; the ephyrae are produced in September–October and the mature adults appear in March–April [65].

During the years of the bloom, *Chrysaora hysoscella* was abundant in the Southern Adriatic Sea and in the Northern Ionian Sea [69] and it spread also in the Northern Adriatic Sea [70] where, even though scarcely, it was previously recorded [71]. During the spring of 1989, coastal blooms of this jellyfish were observed in the Gulf of Trieste [72]. Along the Ligurian Riviera *Chrysaora hysoscella* sightings accounted for around 3.5% of total jellyfish [73]; furthermore it was–with *Rhizostoma pulmo–*the only medusa recorded in Mersin Bay–Eastern Mediterranan Sea (Turkey) [74].

Three different types of nematocysts of *Chrysaora hysoscella* were observed after separation from tissues by using Percoll, but an exhaustive description was not given [75]. Few data are available about the toxicity of *Chrysaora hysoscella* [72,75,109,121] and, to our knowledge, before the 1990s only one report of cutaneous lesions caused by this jellyfish in subtropical waters was available [122]. In spite of this, *Chrysaora hysoscella* is a really dangerous jellyfish, mainly because it has a wide wounding surface, with long tentacles and a large umbrella [65].

Dermatitis with itching and burning due to contact with *Chrysaora hysoscella* were observed within 20 minutes from contact; within few hours they spontaneously disappeared [123]. In the period May–August 1997, 90 human injuries due to jellyfish stings were reported in the zone of Grado (Gulf of Trieste, Italy) and contemporaneous samplings showed *Chrysaora hysoscella* was the only dermotoxic jellyfish constantly present during the considered period [124].

A fraction obtained from this jellyfish was evaluated for dermotoxicity on 25 volunteers by means of scratch-patch test; five of them reacted with itching, erythema and edema after 48 hours [75]. In another study, volunteers experienced itching and burning within 40 seconds after the contact and developed erythema and vesicles after three minutes [72].

Research carried out on a hemolytic fraction of the venom from *Chrysaora hysoscella* showed that this fraction has at least a partial proteinaceous nature with the presence of a cationic protein [75].

The hemolytic activity of the venom of *Chrysaora hysoscella* was evaluated on mouse, sheep and human red blood cells using the dialysed supernatant obtained from the centrifugation of the fluid discharged from the nematocyst, which caused remarkable hemolysis of mouse erythrocytes while sheep and human red blood cells were less sensitive; nevertheless, the sample lost 90% of its hemolytic activity after 24 hours both at room temperature and at −20 °C, and 50% at −36 °C [75].

Recent studies showed that crude extract from *Chrysaora hysoscella* induced 55% mortality with 0.14 μg/μL IC50 in cultured keratinocytes at the dose of 0.15 mg/mL proteins when assessed with neutral red assay; also the mitochondrial activity was highly affected and MTT test showed very high toxicity of crude venom with an IC50 of 0.0019 μg/μL proteins with no subsequent worsening after increasing of venom protein dose. The observed ‘plateau’ effect at high doses of extract has been related with the inhibition of the activity of mitochondrial dehydrogenase [121].

Thus, notwithstanding the scarce poisonousness of this species, from the examined papers it can be possible to state that *Chrysaora hysoscella* nematocysts have a dermotoxic and a cytotoxic activity, even though the substance (or the substances) involved are to date not known. On the whole, Del Negro *et al.* [75] conclude “*Chrysaora hysoscella* is not innocuous and the absence of previous reports on its toxicity might be due to its sporadic presence”. A precautionary conclusion can be read in Del Negro *et al.* [72] who state “aggregations during the holiday season might involve a risk to public health”.

### 2.3. *Pelagia noctiluca* (Forsskål, 1775)

*Pelagia noctiluca*, the “mauve stinger”, is a phosphorescent light pink jellyfish with violet spots. The bell is hemispherical and lenghtened; its marginal edge is divided into 16 lappets and carries eight tentacles [125]. Four oral arms are present beneath the bell. The movements and vertical migrations are affected by light: the pulsations of umbrella were seen to increase remarkable with light decrease [126]. *Pelagia noctiluca* reproduces during late autumn, when adult specimens reach the sexual maturity; the ephyrae appear in winter and the young medusae (2 cm diameter) can be found in early spring. Adults die immediately after the reproduction [65].

*Pelagia noctiluca* is the most dangerous autochthonous Mediterranean jellyfish and was widely studied during and after the jellyfish bloom in the Mediterranean occurring from the late 1970s to the early 1980s [9,10]. Even though, before 1976 in some sea zones such as in the Adriatic Sea this jellyfish was exceptionally recorded [77] its occurrence was notable during all bloom period being also the medusa which mainly characterized the phenomenon in the whole Mediterranean basin [9,10].

The seasonal occurrence of adults and ephyrae, the size structure of the population, the growth and mortality patterns of *Pelagia noctiluca* in the Northern Adriatic Sea were exhaustively studied [127]. Massive outbreaks of *Pelagia noctiluca* have been recorded at intervals of 12 on average and a modelization of the population dynamics showed the maturation at an early stage and the occurrence of small medusae are the main factors affecting the density of this jellyfish [128]. In some zones of the Eastern Mediterranean, such as in Egypt waters, only irregular invasions were recorded [98]. In Tunisian coastal waters *Pelagia noctiluca* is reported to be normally frequent from November to May–when it dominates the population of scyphozoan jellyfish–and disappears in spring; outbreaks were recorded mainly during 1993 and 1994 [87].

During the bloom years fishery was highly affected by jellyfish occurrence which caused also trouble and health problems to fishermen. In addition, the abundance of *Pelagia noctiluca* and the weight of its biomass caused direct damage to fishing because in several cases it was impossible to separate the medusae from fishes, the yield of nets was impaired and the mechanical structures and engines were subjected to notable efforts; in some spring months the weight of jellyfish was even higher than that of catch fish [129,130]. This species was also responsible of disturbance to aquaculture operations causing mortality of reared fishes [88]. Recently a detailed description of the distribution, ecology and toxicity of this species has been given [131].

The lipids and phospholipids in tissues of *Pelagia noctiluca* were evaluated observing that the content of free fatty acids is remarkably high [104]. This research showed that the total lipid content of *Pelagia noctiluca* is comparable to that reported for the highly stinging medusa *Chrysaora quinquecirrha*, while in “non stinging shyphozoan species”, such as *Aurelia aurita*, the total lipid content was only 0.02%; this result induced the Authors to state that “there is a correlation between the lipid armature of tentacles and the intensity of some of their specific bioactions” [104].

*Pelagia noctiluca* is a strong stinging jellyfish and is able to reconstitute its stinging battery within few days after discharge [59]; its venom can produce erythema, edema and vesicles as well as persisting pain in the stung skin [132,133]. Nevertheless, the damage by *Pelagia noctiluca* stings is generally neither severe nor prolonged; the systemic symptoms are extremely uncommon [134], even though several inconveniences such as pain, distress, generalized allergy, bronchospasm, dyspnoea, pruritus, urticaria-like lesions and hyperpigmentation were reported [135]. For the latter aspect, seems that it could be the result of post-inflammatory events or of the tattooing of the pigment of *Pelagia noctiluca* into the skin [136]. Sensitive subjects can suffer damage owing to immunological and/or toxic mechanisms [137,138].

Five different morphological types of nematocysts were recognized in *Pelagia noctiluca*: heterotrichous microbasic euryteles, heterotrichous isorhizas, holotrichous O-isorhizas, atrichous a-isorhizas, and an undescribed type which resembles microbasic p-mastigophores [76]. Recent studies have rearranged the classification of the nematocyst of *Pelagia noctiluca* into three groups [139].

*Pelagia noctiluca* stings produce circinate or irregularly shaped lesions [135] able to cause blemish [134,140] and to induce recurrent eruptions even without further envenomation [133,141]. Patients having significant IgG amounts against crude venom of *Pelagia noctiluca* were seen to show cross-reaction against the venom of *Physalia physalis*[142].

During the jellyfish bloom in the Mediterranean Sea, a lot of subjects were stung by *Pelagia noctiluca,* which occurred often in swarms particularly in the Northern Adriatic Sea and in Greek waters [77,143–145]. The results of epidemiological studies showed the problem was quite extended, but most of cases showed only local symptoms such as redness, pain, itching, burning and vesicles; a minority suffered from severe general symptoms such as dizziness, vomiting, hypotension, diarrhoea, shock [146–148]. Severe stinging events caused by *Pelagia noctiluca* with over than 59,000 treatments were reported in a recent review [88].

The toxicity of *Pelagia noctiluca* was also studied in the laboratory: one of the major problems encountered by researchers was to isolate and to obtain pure suspensions of nematocysts devoid of tissue debris; several attempts were made by soaking tissues in distilled water, centrifugation in saccharose solutions and lyophylization [149], Ficoll, Methylcellulose and discontinuous density gradient centrifugation of Percoll [139], thiocyanate (SCN^−^) ions and heat dissociation [150]. The best results were obtained by Percoll and SCN^−^ which yielded sufficient undischarged nematocysts and a satisfactory isolation; it was stated also that the nematocyst preservation is greatly improved by freezing and by neutral values of pH [151]. Anyhow, as for other Cnidaria [113,152–157], also tissue components of *Pelagia noctiluca* could exert toxicity [158].

The venom of *Pelagia noctiluca* is mainly composed of proteins stabilized by Ca^2+^ ions [138,144,159]; glutamic acid amounts for 80% of the proteins of the capsule fluid and 90% of the capsule wall [159]. The capsular fluid was partially purified and fractionated and the molecular weight was determined: the toxic activity was ascribed to presumably proteinaceous macromolecules with 44–66 kDa MW [160]. Also the observed absence of effects on cell DNA confirmed the proteinaceous nature of the venom [158].

Preparations of intact nematocysts and fractionated capsule fluid from *Pelagia noctiluca* were tested in laboratory experiments; intact nematocysts caused erythema on mice skin both after simple contact and intradermal injection; oedematous and nodular/necrotic phenomena with leukocyte infiltrate were also pointed out [132]. The fractionated fluid was assayed on heart activity of rats and on neuromuscular activity of frogs showing scarce activity on cardiac frequency and remarkable toxicity on neuromuscular synapses [160]. Partially purified crude venom from *Pelagia noctiluca* was seen to have cardiotoxic activity against cultured chick embryo cardiocytes [161].

Experiments carried out by employing human volunteers showed that nematocyst preparations caused local symptoms in less than 50% of treated subjects who developed erythema and pruritus after 30 minutes which persisted for 2–3 days [132]. The venom of *Pelagia noctiluca* was seen to have high antigenic potential for man [162] and to cross-react with monoclonal antibodies to *Physalia* and *Chrysaora*venoms both experimentally [161] and clinically [138].

Cytotoxicity tests have shown that the crude venom from *Pelagia noctiluca* can remarkably affect the survival and growth of mammalian cells in culture [148,158] even though its activity seems to be lower, both after short- and long-term treatments, than that of other extracts from apparently less venomous jellyfish [148,155,157,163]. Furthermore, the crude venom was able to induce ATP increase in treated cells [158] and remarkable hemolysis of chicken and rabbit erythrocytes after freezing (−20 °C and −80 °C) and after lyophilization, but showed a scarce effect on fish red blood cells [164].

Recent studies [165] evaluated the effect of osmotic protectants such as carbohydrates, cations (Mg^2+^, Ca^2+^, Ba^2+^, Cu^2+^, K^+^), proteases (collagenase, trypsin, [alpha]-chymotrypsin, papain) and antioxidants against the hemolysis induced by the venom of *Pelagia noctiluca*, showing that the inhibition of such substances depends on their molecular weight and is more pronounced by using Ba^2+^ and Cu^2+^ and less with oxidants, while proteases do not seem to have a significant effect. The Authors suggest the hemolysis induced by the crude venom of *Pelagia noctiluca* could be due to a pore-forming mechanism rather than to oxidative damage of the cell membrane.

The free radical scavenger and antioxidant melatonin was used in the therapy of rats experimentally stung with crude venom of *Pelagia noctiluca* and suffering from acute paw inflammation characterized by lipid peroxidation and occurrence of polymorphonuclear neutrophils; the venom was observed to promote the expression of inducible nitric oxide synthase and the activation of the nuclear enzymes and the administration of melatonin reduced remarkably the inflammation [166].

The pore forming mechanism on cell membranes was suggested to explain the cell lysis of red blood cells induced by *Pelagia noctiluca* venom; nematocytes from the anthozoan *Aiptasia mutabilis* treated with *P. noctiluca* venom did not show lysis nor cell volume regulation capability, suggesting an inhibitory effect on cell membrane transport mechanisms [167].

The crude venom of *Pelagia noctiluca* was seen to block the discharge of acontia from *Calliactis parasitica*, with a dose-dependent irreversible effect; it was hypothesized that the crude venom could act blocking the junctions involved in the communication between cells, damaging the nematocyte-activating cells or inhibiting the Ca^2+^ influx needed for the triggering of the discharge [168].

### 2.4. *Cotylorhiza tuberculata* (Macri, 1778)

*Cotylorhiza tuberculata* (“fried egg jellyfish”) is a brown to yellowish medusa with a flattened bell with a raised central portion. The marginal bell is divided in 16 main lobes further subdivided into numerous small lobes. It carries eight oral arms with several projections having violet or purple tips [12].

*Cotylorhiza tuberculata* is considered to be an endemic species of the Mediterranean Sea, but it can be found also in the Red Sea and near the Canary Islands [87]; adult medusae disappear during the colder months while the scyphistomae are thought to survive and strobilate in spring when the temperature increases [169]. Specific indications report that it occurs normally in the Ligurian Sea in summer [170], while it is uncommon in the Tunis Bay and occurs in significant numbers only in October and in November [87]. The occurrence of this jellyfish in the Eastern Mediterranean is also limited, but a large swarm was sighted along Israeli coasts during the bloom years [79].

Swarmings of this jellyfish with an impressive bloom in spring-autumn 1921 were reported in the Northern Adriatic [71]. During the jellyfish bloom of the 1970s–80s, a generalized increase of *Cotylorhiza tuberculata* was recorded [107]; great quantities were seen both in open sea and along the coasts in the Northern Adriatic Sea [77]; it was also one of the most occurring jellyfish in the Southern Adriatic and in Northern Ionian Sea [69]. Furthermore, coastal aggregations of *Cotylorhiza tuberculata* were observed abundantly along Maltese coasts [80]. Along the Ligurian Riviera, *Cotylorhiza tuberculata* sightings amounted to around the 5% of the total from 1984 to 1985 [73].

Eutrophication processes induced by nutrient loading were indicated to be indicators of summer proliferation of *Cotylorhiza tuberculata* in the Mar Menor lagoon (Southeast Spain) [81,82]; the jellyfish number in the lagoon (considering both *Rhizostoma pulmo* and *Cotylorhiza tuberculata*) was estimated to be around 47 million [81], resulting in serious inconveniences to tourism and to local commercial fisheries with consequent serious economic repercussions [83]. In spite of these problems, it was stated that a top–down control by large gelatinous zooplankton colonizing the lagoon occurred, producing a new equilibrium in which the proliferation of jellyfish could have been acting as an eutrophication controlling agent [83,84]. The occurrence of jellyfish was considered a symptom of environmental changes and a plague in the lagoon, affecting tourism and inducing the regional Government to establish annual capture efforts [85]. Notwithstanding these problems, it was stated that *Cotylorhiza tuberculata* did not clog the fishing nets entirely [77].

Recent studies identified in *Cotylorhiza tuberculata* eurytele and birhopaloid (type II) nematocysts, having broad and prominent shafts [33]. From the toxicological point of view, *Cotylorhiza tuberculata* was defined as “not very toxic” [144] and “unharmful species causing troubles to bathers when it occurs copiously” [107]; as a matter of fact, large swarmings of *Cotylorhiza tuberculata* were reported to cause nuisances in bays and in coastal areas of Greece in summer [169]. Furthermore, as stated above, cospicuous occurrence of *Cotylorhiza tuberculata* recently caused organizational problems in Spanish waters alarming the Authorities by the potential decrease of tourism [116].

### 2.5. *Rhizostoma pulmo* (Macri, 1778)

*Rhizostoma pulmo* is a large and heavy whitish Mediterranean jellyfish with violet edges. The umbrella is hemispherical and bell-shaped and lacks tentacles; several marginal lobes are present.

The occurence of this jellyfish increased remarkably during the years of the bloom (from the late 1970s to the early 1980s) and its presence, even though less harmful in comparison to *Pelagia noctiluca*, caused many economic problems and also health implications.

Owing to its weight, *Rhizostoma pulmo* is able to break fishing nets (it was reported that in some spring months the weight of medusae was higher than that of taken fishes); in addition, it can impair fishery net yield reducing the available surface for fishing, being impossible to separate the medusae from caught fish [129,171]. In spite of this, *Rhizostoma pulmo* was not considered exaggeratedly detrimental for fishing because due to its size and consistency it doesn’t completely clog the nets [77].

During the years of the bloom in the Mediterranean Sea, *Rhizostoma pulmo* occurred in large numbers in the Northern Adriatic Sea, in open sea and along the coastline [77], as well as in the Southern Adriatic Sea and the Northern Ionian Sea, mainly in winter [69]. Along the Ligurian Riviera, from 1984 to 1985, *Rhizostoma pulmo* was sighted in a wide sea area and amounted to nearly 7% of total sighted jellyfish [73]. In Turkish waters (Mersin Bay–Eastern Mediterranan Sea) during the years of the bloom, not many *Rhizostoma pulmo* were present, however the population of medusae was composed only of this species and *Chrysaora hysoscella*[74].

*Rhizostoma pulmo* was indicated to be the largest and most abundant jellyfish in Lebanese coastal waters, occurring usually in late spring when the temperature increase up to 25.5 °C, staying in Lebanese waters up until mid-August and disappearing later on; swarms seemed to correlate with high temperature and nutritional factors connected to the abundance of zooplankton, which is the food for this microphagous jellyfish [86]. During the last decade, in some Eastern Mediterranean waters *Rhizostoma pulmo* has been replaced by *Rhopilema nomadica* [89–91].

This jellyfish is common in the Black Sea [66] and is constantly present also in Tunisian waters (Gulf of Tunis), where in May, September and October the maxima of density (0.003 ind/m^3^) of 20–40 cm umbrellar size individuals has been recorded [87].

Recent proliferations of *Rhizostoma pulmo*, presumably connected to nutrient influx and eutrophication processes, have been recorded in the Western Mediterranean (Mar Menor lagoon–Southeast Spain) where they are allochthonous [82]; these proliferations caused serious inconveniences to tourism and to local commercial fisheries with heavy economic repercussions [83]. Studies by Pérez-Ruzafa *et al*. [81] estimated 0.45 individuals per 100 m^3^ in the lagoon and indicated that the massive proliferation of jellyfish in the Mar Menor could have generated a new equilibrium in which jellyfish act as an agent controlling the consequences of the eutrophication [83,84]. In any case, *Rhizostoma pulmo* caused organization problems in Spanish waters because the Authorities became alarmed about the potential decrease of tourism [116] and were induced to organize jellyfish capture plans [85].

*Rhizostoma pulmo* is moderately venomous, so it is not a strong stinging species; therefore it is commonly considered innocuous and completely harmless to humans. Notwithstanding these characteristics, it was indicated to cause burning to fishermen in the Adriatic Sea [129,171] and in Lebanese waters it frequently caused damage to fishermen and to swimmers who suddenly complained of damage after contact [86]. Specific studies have shown that *Rhizostoma pulmo* has four types of nematocysts, which according to the classification of Mariscal [8] were indicated to be heterotrichous microbasic euryteles, holotrichous isorhizas, atrichous a-isorhizas and atrichous-isorhizas [76].

The contact with *Rhizostoma pulmo* may cause erythematous and ulcerous lesions [75]; rare cases of dermatitis are described as slight erythemas disappearing spontaneously after a few hours, even though burning on the skin and particularly the lips, sneezing and rhinorrhea, urticaria and systemic symptoms have been referred [172]. Episodes of contact dermatitis were reported recently after *Rhizostoma pulmo* stinging, confirming its toxicity to humans: the contact was reported to cause immediate cutaneous pain followed by erythematous slightly infiltrated eruption and formation of vesicles; after local corticosteroid therapy the pain disappeared within 36 hours [172].

The venom of *Rhizostoma pulmo* was reported to be not of protein nature because it does not provoke immune phenomena [173] and loses 50% of its hemolytic activity when stored for one month at −20 °C [174]. A very large hemolytic protein (cytolysin), named Rhizolysin, having a molecular weight of approximately 260,000 Da and a sedimentation coefficient of 10.3 S has been isolated from the nematocysts of *Rhizostoma pulmo*; Rhizolysin seems does not have phospholipase A activity, acts at a optimum pH of 6.75 and is completely inhibited by sucrose and less by cholesterol and sphingomyelin [174].

The peaks obtained through chromatographic (HPLC) separation of crude extracts from *Rhizostoma pulmo* appear similar to those from other pelagic jellyfish and differ mostly from those from benthic Cnidaria, indicating an analogy between species having similar ecological and environmental arrangement [175]. Further research, which evaluated the cytotoxicity of low molecular weight (<12 KDa) components obtained through chromatographical (HPLC) separation, from oral arms in toto showed pronounced lethal effects on cultured V79 cells suggesting that the venom of *Rhizostoma pulmo*, as that of other Cnidaria, consists of several differently toxic fractions [176].

The crude toxin of *Rhizostoma pulmo* evidenced stronger cytotoxic effects on V79 cells than that of other Mediterranean jellyfish and anemones both after short-term (1–3 hours) and long term (five days) treatments, greatly affecting cell survival and growth (doubling) rates [148] and causing a decrease of ATP levels with complete depletion of ATP after 115 minutes of treatment with 150,000 nematocysts/mL[177].

The tissues of *Rhizostoma pulmo* sampled from oral arms and devoid of nematocysts were found to have a strong cytotoxic activity on cultured cells and showed IC50 values ranging from 16.9 to 49.9 μg proteins/mL according to the origin of the tissue (from the external to the internal part of the oral arm, respectively); this indicates that also jellyfish tissues contain compounds that highly affect the viability of treated cells [163]. Subsequent studies showed that at the concentration of 37.6 μg/mL, the proteins from tissues devoid of nematocysts produced the death of 50% of treated V79 cells and a strong hemolysis of human eryrthrocytes, but were not clastogenic for human lymphocytes - these did not show any increase of micronuclei induction [157].

### 2.6. *Rhopilema nomadica*(Galil, 1990)

*Rhopilema nomadica* has a nearly spherical icy-blue-colored umbrella, thickest centrally and minutely granulated externally, whose margins are divided into 64 rounded lappets; from arm disc sides eight pair of large scapulets, distally provided with long filaments, arise [79]. The bell size of this jellyfish can range from 10 to 80 cm in diameter and the whole medusa can have a weight of 40 Kg [92].

This jellyfish is commonly known as “lessepsian”, a term which indicates the species that started to migrate from the Red Sea to the Mediterranean Sea after the opening of the Suez Canal in 1869; an event that connected the Mediterranean Sea to the Red Sea and, through the latter, to the Indian and Pacific Oceans. The opening of the canal unintentionally joined two biogeographical provinces [93] and the passage of species, mainly colonizing the Mediterranean from the Red Sea, have had a remarkable effect on bio-ecology and on ecosystem balances [90].

*Rhopilema nomadica* is an Indopacific scyphozoan, which first appeared along the coastlines of Israel in 1977 [90] and to date is found along all the eastern Mediterranean [88,94]. Large aggregations of this jellyfish were observed starting from the 1980s along Israeli coasts [92] and annual blooms occurred during summer from 1989 to 1992; afterwards *R. nomadica* rapidly spread in the eastern Mediterranean Basin migrating towards Lebanon and Syria [95]. Its spreading in Turkey waters was studied in relation to physico-chemical characteristics of water in eastern, north-eastern Mediterranean and Iskenderun Bay [96]. The proliferation of *Rhopilema nomadica* in the eastern Mediterranean caused profound changes in the indigenous biota [93] such as the replacement of *Rhizostoma pulmo* in Israeli Mediterranean waters during the last decade [89–91].

As concerns the reproduction, the life cycle from planula to ephyra to young medusa was described [92]. The strobilation of polyps was observed to be dependent on temperature: at temperature below 16 °C polyps do not strobilate, while rapid strobilation occurs between 18 and 20 °C; the process declines again at 24–26 °C; therefore, the spring rise of water temperature supports the strobilation, which on the contrary is inhibited in winter and in summer [95]. On the basis of the observed sensitivity of polyps to low temperatures, it was suggested that the dispersal of *Rhopilema nomadica* could be limited to the eastern Mediterranean [95].

The nematocysts of *Rhopilema nomadica* were studied using light and electron microscopy and three morphological types were identified: heterotrichous isorhiza haploneme, holotrichous isorhiza haploneme and heterotrichous microbasic eurytele; the latter is typical of scyphozoans and was recognized as similar to those from *Pelagia* and *Rhizostoma* [97].

This species, existing in huge numbers, poses a big threat to humans producing burning, pain and redness of the affected skin that is counteracted by topical treatment with vinegar. The encounters with *Rhopilema nomadica* produced painful stings to bathers and fishermen in a large area of the Eastern Mediterranean Sea from Egypt to Turkey [178]. Despite this, rare systemic reactions with severe delayed skin reaction have been observed: as described [179], two days after contact with the jellyfish a patient developed severe burning, pain and itching at the affected skin areas with marked erythema, papulovesicular eruptions and urticaria-like eruptions that worsened with sun exposure. Such delayed reactions were reported also one week after envenomation [180]. Nevertheless, severe reactions have been reported when eyes are affected, resulting in conjuctivitis, chemosis, and eyelid oedema [181].

Preparations from nematocysts of *Rhopilema nomadica* were tested with a scratch-patch test on volunteers who had come into contact with this jellyfish at some point in time, and were shown to cause dermatotoxicity with mild erythema after 48 hours (particularly extended in one case), itching and severe burning in one case [182].

The venom of *Rhopilema nomadica* was seen to have e-chymotrypsin-like serine protease activity, phospholipase A2 activity and temperature-dependent haemolytic activity [178]. The purification of a new polypeptide toxin from tentacles of *Rhopilema nomadica* was reported, indicating that, based on the amino acid sequence of its N-terminal segment, it is a phospholipase A2 toxin that resembles some vertebrate (reptile) and invertebrate (hymenopterous) venoms [183]; Phospholipase A2 are enzymes present in several cnidarian species, which may be involved in physiological cell membrane lipid metabolism, in the irritation at the stinging site, in the systemic envenomation syndrome and in the enhancement of the haemolytic activity [112, 114]; they may have a role in the capture and digestion of prey and in the defense of the animal [112]. The characteristics of this toxin may explain the local dermonecrotic and the cardiac and respiratory events after envenomation [183]. Antibody analysis through immunocytochemical approach allowed to found the site of toxin allocation in nematocysts showing that it is stored “on the outer (‘cytoplasmic’) surface of the inverted tubule folded in the capsule of the resting nematocyst”; furthermore, mechanical forces drive the transfer of the polypeptide because “during discharge the toxin is translocated to the internal surface surrounding the lumen of the everting tubule, and its delivery (…) is apparently propelled by the high hydrostatic pressure of the capsule” [183].

*Rhopilema nomadica* has been recently indicated to be the responsible for veliger mortality and reduced recruitment of the predatory whelk *Stramonita* (=*Thais*) *haemastoma* [184]. In addition to the concern for its stinging, this species is considered to be also damaging to fishing activities; in fact, clogging of fishing nets was reported during the early 1990s [88,185]. It also caused economic loss affecting tourism and blocking the filters of seawater drawing systems [90].

The mesoglea from *Rhopilema nomadica* was seen to be a suitable support for attachment and spreading of cells of anthozoan (sea anemones, scleractinian coral and alcyonacean corals) cells *in vitro* when compared with other artificial and organic substrata (including known components of cnidarian mesoglea such as fibronectin and collagen) routinely used in vertebrate and invertebrate cell culture; perhaps this was due to the composition and morphology of the intact substrate because neither isolated artificial and organic components nor mesoglea extraction were effective to support cell attachment [186]. These interactions between cells of Anthozoa and mesoglea of Scyphozoa could be useful for the development of cnidarian cell cultures.

### 2.7. Other scyphozoan jellyfish less frequently recorded in the Mediterranean Sea

#### 2.7.1. *Nausithoe punctata* (Kölliker, 1853)

*Nausithoe punctata* is a small coronate scyphozoan jellyfish with 2 cm maximum bell diameter [12]. It has been indicated as a “primitive scyphomedusa” and the ultrastructure of its spermatozoa was studied [187]. It is a known species in the Adriatic Sea [144], but it is quite unfrequent; usually it occurs from December to May in surface Northern Adriatic waters [4]. Ephyrae, young and adult specimens were observed sporadically in the Adriatic Sea from 1974 to 1985 [188]. During and before the jellyfish bloom it was recorded in winter and in spring in Egypt waters off the coast of Alexandria [98].

#### 2.7.2. *Cassiopea polypoides* (Keller, 1883)

*Cassiopea polypoides* is an Indo-Pacific species; the small aggregations found in 1987 in neritic Lebanese waters were the first record of this jellyfish in the Mediterranean Sea [86].

It was reported that the eutrophication may increase the biomass of *Cassiopea* spp. thanks to particular adaptations which allow these jellyfish to survive under eutrophic conditions [99].

#### 2.7.3. *Cassiopea andromeda* (Forsskål, 1775)

*Cassiopea andromeda* is distributed in the Red Sea and in the Indo-Pacific Ocean; in the Mediterranean Sea occurs along Lebanon and Israeli coasts coming from the Suez Canal [79]. *Cassiopea andromeda* was the first known lessepsian and the first Erythrean scyphozoan jellyfish found in the Eastern Mediterranean after the opening of the Suez Canal [189,190]. It was reported into the Suez Canal already in the late 19th century; the first record in the Mediterranean was from Cyprus [191]. Subsequently, it was occasionally reported in some areas of the Eastern Mediterranean as far as in the Aegean Sea [190].

*Cassiopea andromeda* is a venomous species and its nematocysts have been studied. The crude venom produced pain when applied to human lips, resulted in lethalilty to mice at a dose of 0.21 mg protein kg^−1^ mouse and 50 μg protein caused vasopermeability and dermonecrosis after injection into mouse skin; furthermore, crude venom was shown to have phospholipase A2 activity and to induce lysis of mouse lymphocytes. Dosages of 1 μg protein lysed 50% of treated human erythrocytes [62].

#### 2.7.4. *Discomedusa lobata* (Claus, 1877)

The occurrence of this medusa was recorded in Egyptian coastal waters [98]. *Discomedusa lobata* is considered occasional in the Mediterranean; histological and cytological research were carried out on gonads founding a para-ovular body similar to that of *Pelagia noctiluca* [192].

#### 2.7.5. *Phyllorhiza punctata* (von Lendenfeld, 1884)

It is distributed in Australian, Philippine and Japanese waters; In the Mediterranean Sea only one record was reported in Israeli waters [79].

## 3. Conclusions

Plants and animals are a source of extracts providing therapeutic activity from which several organic substances have been isolated and some of them currently have application as compounds of biomedical interest such as drugs, pigments, insecticides, *etc*. or were used to synthesize biologically active molecules. The biotoxins are included among these compounds and can have different pharmacological properties when used at sublethal doses.

The study of natural compounds and of venoms and associated biotoxins have had an impressive advance during the last decades and a number of marine organisms, such as marine Cnidaria, have been recognized as a potential and promising source of bioactive compounds useful for several purposes. As a matter of fact, some representative species of this *phylum* are to date considered as producers of substances that could have an application in human pathology.

In the Mediterranean countries, studies concerning the biology and the ecology, as well as the toxicology, of Cnidarian jellyfish, have noticeably increased during the last decades as a result of the blooms that happened in several coastal and pelagic areas. These phenomena, and the sanitary and economic implications they caused, outshined the previous disinterest for the study of the poisonousness of Mediterranean Cnidaria owing to the generalized weak harmfulness of most of species living in this area. This is to date a current topic because of large swarmings and strandings of jellyfish, particularly *Aurelia aurita* and *Pelagia noctiluca*, occurring recently in several Mediterranean coastal waters as, for example, into the Gulf of La Spezia (Eastern Ligurian Sea) as reported by professional fishermen.

The better knowledge of these organisms has also allowed to identify new aspects about the functioning of their morphological structures and the activity of compounds extracted from their nematocysts and tissues. The emphasis of these studies is justified also by the needing of innovative systems able to counter the activity of venoms. The applicative research led to the production of tools for the management and the protection from the damage they could exert, such as the making of sunlight protection products simultaneously protecting from Cnidarian stinging, containing appropriate components isolated from clown fish, which are, as a matter of common knowledge, not injured by nematocysts [193,194]; the inhibitor of stings is hydrophobic, thus counters notably the contact of nematocysts with the skin, contains glycosaminoglycans similar to those of jellyfish bell, activates the self-recognition system of the jellyfish and, through calcium and magnesium it includes, blocks the transmembrane signaling channels of jellyfish and reduces the osmotic forces needing for nematocyst firing [193]. Also compounds such as lanthanum sulfate were indicated to provide with protection *in vitro*against jellyfish venoms [195] and were shown to block the venom-induced 45Ca uptake [196].

In conclusion, all species of jellyfish in the Mediterranean are capable of some minor injury, however, most are relatively harmless. *Pelagia noctiluca* and *Chrysaora hysoscella*, as well as the lessepsian *Rhopilema nomadica*, are considered the most venomous; however, considering the widespread abundance of *Pelagia noctiluca*, it is by far the biggest cause of jellyfish stings in the Mediterranean. Thus, the study of not-highly-poisonous jellyfish, such as the Mediterranean species, could be important to develop research models and to detect compounds which could have an application as bioactive substances. Further research is needed to probe the effective possibilities of Mediterranean jellyfish in this field.

## Figures and Tables

**Table 1:** Data summarizing the occurrence, envenomations (+ = infrequent; ++ = frequent; +++ = very frequent), poisonousness (+ = scarce; ++ = middle, +++ = quite high) and morphological types of nematocysts of the main Mediterranean scyphozoan jellyfish. The numbers in brackets correspond to the references. (*) in scyphistomae polyps. (**) reports from non-Mediterranean zones show variable stinging potency [62–64].

Species	Occurrence in the Mediterranean Sea	Cases of envenomation	Poisonousness	Described nematocysts
*Aurelia aurita*	whole Mediterranean Sea [65], whole Black Sea [66] (mainly coastal)	+ (**)	+ (**)	heterotrichous microbasic eurytele [67,68] (*); atrichous isorhiza (“a” atrichs, oviform polyspiras) [67,68] (*); heterotrichous microbasic rhopaloid [45] (*) homotrichous isorhiza haploneme [45] (*) heterotrichous microbasic eurytele [45] isorhiza with subsperical capsule [45]
*Chrysaora hysoscella*	mainly Adriatic Sea [69,70–72], Ionian Sea [69], Ligurian Sea [73], Eastern Turkey coasts [74]	+	++	three undescribed types [75]
*Pelagia noctiluca*	whole Mediterranean Sea [9,10]	+++	+++	heterorichous microbasic eurytele [76]; heterotrichous isorhiza [76]; holotrichous-a-isorhiza [76]; atrichous-a-isorhiza [76]; undescribed type [76]
*Cotylorhiza tuberculata*	whole Mediterranean Sea [69,71,73,77–85]	none	no	euryteles [33]; birhopaloid (type II) [33]
*Rhizostoma pulmo*	mainly Adriatic Sea [69,71,77], Ionian Sea [69], Ligurian Sea [73], Eastern Mediterranean [74,86], Tunisian waters [87] Western Mediterranean [81–85] Black Sea [66]	++	++	heterotrichous microbasic eurytele [76]; holotrichous isorhiza [76]; atrichous-a-isorhiza [76]; atrichous isorhiza [76]
*Rhopilema nomadica*	Eastern Mediterranean [88–96]	++/+++	++/+++	heterotrichous isorhiza haploneme [97]; holotrichous isorhiza haploneme [97]; heterotrichous microbasic eurytele [97]
